# BUT WHO LOOKS AT ME? About a daily clinical case in treatment in a mental health center

**DOI:** 10.1192/j.eurpsy.2024.1355

**Published:** 2024-08-27

**Authors:** B. Gamo Bravo, M. E. Gonzalez Laynez, S. M. Bañon Gonzalez, N. Ogando Portilla

**Affiliations:** ^1^psichiatry, University hospital infanta Sofia, San Sebastian de los Reyes; ^2^psichiatry, University hospital of Toledo, Toledo, Spain

## Abstract

**Introduction:**

BUT WHO LOOKS AT ME?

Patient around thirty years old, teacher and with obsessive, anxious, paranoid, schizotypic semiology that affects his functionality to the point of isolation, and take sick leave, which with pharmacological treatment with antipsychotics such as aripiprazole and olanzapine and the antidepressant sertraline (at a final dose of 200 mg) and group psychotherapy in multifamily groups remits from these symptoms with functional and symptomatic improvement.

**Objectives:**

Highlight the diagnostic difficulties due to the coexistence of symptoms that are part of personality imbalances or first-order diagnostic entities as in this case, depressive picture in a personality with obsessive and paranoid traits

**Methods:**

Describe the evolution and psychiatric clinical decompensation of a patient with depression and anxiety and a personality of cluster A traits, paranoid type and obsessiveness

**Results:**

CLINICAL DIAGNOSTIC TRIAL

ANXIOUS DEPRESSIVE SYNDROME (PREDOMINANCE OF SYMPTOMS OF OBSESSIVENESS AND DISTRUST)

MIXED CLUSTER A PERSONALITY DISORDER (PARANOID AND SCHIZOTYPIC TRAITS)

**Conclusions:**

**Discussions and conclusions:** There is a gap difficult to separate in many cases between obsessiveness and paranoidism as communicating vessels, whose worsening of one worsens another and whose improvement of one leads to the improvement of the other, which at the pharmacological level respond to combined approach versus potentiated atypical antipsychotics and antidepressants such as sertraline that help us neutralize the discomfort

**Disclosure of Interest:**

None Declared

